# Synergistic Effect of a Combination of Proteasome and Ribonucleotide Reductase Inhibitors in a Biochemical Model of the Yeast *Saccharomyces cerevisiae* and a Glioblastoma Cell Line

**DOI:** 10.3390/ijms25073977

**Published:** 2024-04-03

**Authors:** Kirill A. Kulagin, Elizaveta S. Starodubova, Pamila J. Osipova, Anastasia V. Lipatova, Igor A. Cherdantsev, Svetlana V. Poddubko, Vadim L. Karpov, Dmitry S. Karpov

**Affiliations:** 1 Center for Precision Genome Editing and Genetic Technologies for Biomedicine, Engelhardt Institute of Molecular Biology, Russian Academy of Sciences, 119991 Moscow, Russia; kirill007kulagin@gmail.com (K.A.K.); estarodubova@yandex.ru (E.S.S.); osipova.pamila@yandex.ru (P.J.O.); lipatovaanv@gmail.com (A.V.L.); igor.cherdancev.2012@mail.ru (I.A.C.); 2 Engelhardt Institute of Molecular Biology, Russian Academy of Sciences, 119991 Moscow, Russia; karpov@eimb.ru; 3 Institute of Biomedical Problems of Russian Academy of Sciences, 123007 Moscow, Russia; poddubko@imbp.ru

**Keywords:** *Saccharomyces cerevisiae*, glioblastoma cell line, proteasome, ribonucleotide reductase, bortezomib, hydroxyurea, gemcitabine

## Abstract

Proteasome inhibitors are used in the therapy of several cancers, and clinical trials are underway for their use in the treatment of glioblastoma (GBM). However, GBM becomes resistant to chemotherapy relatively rapidly. Recently, the overexpression of ribonucleotide reductase (RNR) genes was found to mediate therapy resistance in GBM. The use of combinations of chemotherapeutic agents is considered a promising direction in cancer therapy. The present work aimed to evaluate the efficacy of the combination of proteasome and RNR inhibitors in yeast and GBM cell models. We have shown that impaired proteasome function results in increased levels of RNR subunits and increased enzyme activity in yeast. Co-administration of the proteasome inhibitor bortezomib and the RNR inhibitor hydroxyurea was found to significantly reduce the growth rate of *S. cerevisiae* yeast. Accordingly, the combination of bortezomib and another RNR inhibitor gemcitabine reduced the survival of DBTRG-05MG compared to the HEK293 cell line. Thus, yeast can be used as a simple model to evaluate the efficacy of combinations of proteasome and RNR inhibitors.

## 1. Introduction

Proteasome is a large multisubunit protease complex involved in the ATP-dependent selective degradation of regulatory proteins and proteins with damaged structures [1]. Proteasome, providing the degradation of regulatory proteins and proteins with a disrupted structure, is necessary for the implementation of all basic functions of a cell and its resistance to stress. The activity of proteasomes is increased in tumor cells [2] due to the active process of the accumulation of proteins with an incorrect structure caused by the accumulation of mutations in genes and impaired quality control of newly synthesized proteins. Proteasome inhibitors such as bortezomib, carfilzomib and ixazomib are used in the therapy of some cancers: multiple myeloma (MM) [3,4], mantle cell lymphoma [5], acute lymphoblastic leukemia [6] and osteosarcoma [7]. There are also clinical trials on the use of proteasome inhibitors in the treatment of brain tumors including glioblastoma (also called glioblastoma multiforme, GBM) [8,9].

GBM is the most aggressive and deadly form of brain cancer. It accounts for ~80% of all primary brain gliomas and ~60% of all adult brain tumors [10]. The typical treatments for GBM are surgical resection of the tumor followed by radiotherapy and temozolomide [11]. GBM is characterized by the spatial and temporal heterogeneity of expression signatures and can change subtypes in response to therapy, leading to the development of recurrent forms resistant to therapy [12]. GBM is associated with poor survival, which is ~14–20 months, and about 5% of patients survive 5 years after treatment [13]. During the therapy of GBM, as in other cancers, selection and further growth of its clones resistant to the action of therapeutic drugs may occur [12,14], which requires a change in the treatment strategy of the disease. Therefore, new, more effective ways of treating GBM are desperately needed. The direction of combinatorial therapy of oncologic diseases is intensively developing, in which at least two active components are used [15,16,17].

Selecting the optimal composition and dose of drugs for combinatorial therapy, even using GBM cell lines as a model, can be a time-consuming and laborious process. We propose to use yeast as a preliminary, simple, rapid model for the research and development of drug combinations for GBM treatment. Yeast has already been used in GBM research [18,19] and for the development of a high-throughput strategy for selection-specific antibodies against GBM stem cells [20]. Using yeast as a biochemical model of tumors, we have previously obtained results indicating that the use of proteasome inhibitors in combination with DNA-damaging compounds may not be effective in tumor therapy [21,22]. In the same model, we have shown that the use of DNA repair inhibitors [22] or ribonucleotide reductase (RNR) inhibitors [23] under conditions of proteasome inhibition can be an effective way to control tumors. 

RNR is a highly conserved enzyme that catalyzes the limiting step of the de novo pathway for the synthesis of deoxyribonucleoside triphosphates (dNTPs), which are used for DNA replication and repair [24]. The enzyme is a heterotetramer in which two large subunits perform the function of catalysis, and two small subunits are responsible for the regulation of complex activity. In yeast and mammalian cells, RNR activity is controlled at several levels by the regulation of gene expression [25,26,27,28], oligomerization of subunits, post-translational modifications of subunits [29,30], subcellular localization [31,32], allosteric inhibition by dNTPs [33] as well as through the degradation of subunits [23,34,35] and regulatory proteins [36,37] in the proteasome or by autophagy [38]. RNR activity has been found to increase in tumors, which is associated with both active tumor cell division and more active DNA repair processes [39,40]. RNR is considered a promising target in combined antitumor therapy. So, high antitumor activity of the combination of bortezomib and 4-hydroxysalicylanilide (a specific inhibitor of the RRM2 subunit of RNR) was shown against MM cell lines, including primary cells obtained from a patient with MM refractory to bortezomib [41]. Recent work shows that drugs that target RNR or inhibit its expression sensitize GBM to standard therapy [42,43,44]. To our knowledge, RNR inhibitors in combination with proteasome inhibitors have not yet been investigated for GBM treatment [12].

Thus, our work is the first to investigate the possibility of using a combination of proteasome and RNR inhibitors to suppress the growth of GBM cells. In the present study, we determined mRNA levels, subunit levels, and RNR activity under conditions of impaired proteasome function. The effects of hydroxyurea and bortezomib alone and together on the survival of a wild-type yeast strain and its mutant derivatives with reduced proteasomal activity and altered RNR regulation were evaluated. In addition, the survival of HEK293 and DBTRG-05MG lines under the action of a combination of bortezomib and gemcitabine was determined.

## 2. Results

### 2.1. RNR Subunit Levels Are Elevated in Strains with Impaired Proteasome Function

**Figure 1:**
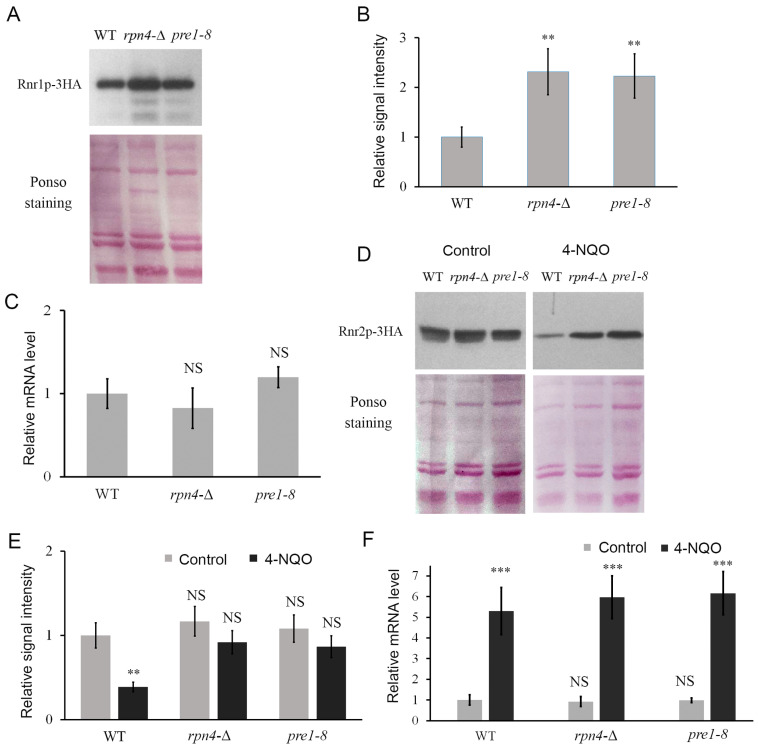
RNR subunit levels are elevated in strains with impaired proteasome function. (**A**) Western blot analysis of the level of 3 × HA epitope-labeled Rnr1p in *rpn4*-∆ and *pre1–8* strains under normal conditions. Ponceau staining was used as a loading control. The intensity of Rnr1-3HA bands was normalized by the corresponding sum of protein band signals obtained by Ponceau staining. (**B**) Quantification of Western blot signal intensity. Band intensities were determined using the ImageJ program. The relative signal of Rnr1p in the wild-type strain was taken as one. Data represent mean ± SD (*n* = 3). (**C**) Relative level of *RNR1* mRNA in *rpn4*-∆ and *pre1–8* strains under normal conditions. The actin gene was used as a reference gene. The signal level in the wild-type strain was taken as one. Data represent mean ± SD (*n* = 3). (**D**) Western blot analysis of the level of 3 × HA epitope-labeled Rnr2p in *rpn4*-∆ and *pre1–8* strains under normal conditions and after 4-NQO-induced DNA-damaging stress. The intensity of Rnr2-3HA bands was normalized by the corresponding sum of protein band signals obtained by Ponceau staining. (**E**) Quantification of Western blot signal intensity. Band intensities were determined using the ImageJ program. The relative signal of Rnr2p in wild-type strain in control was set as one. Data represent mean ± SD (*n* = 3). (**F**) Relative level of *RNR2* mRNA in *rpn4*-∆ and *pre1–8* strains under normal conditions and after 4-NQO action by real-time PCR. Actin served as the reference gene. The mRNA level in the wild-type strain was set as one. Data represent mean ± SD (*n* = 3). Statistical significance of differences between mean values was assessed using Student’s *t*-test. NS—no significant differences (*p* > 0.05), ** means 0.001 < *p* < 0.01, *** means *p* < 0.001.

We have previously shown that Rnr1p is a substrate of the proteasome [23], which is evidenced by an increase in the content of Rnr1p under the action of the proteasome inhibitor bortezomib or in a mutant strain with reduced proteasome activity due to decreased expression of the *PRE1* gene encoding an essential structural subunit of the proteasome (called *pre1–8*). In the present study, Western blot analysis showed that in the mutant strain *rpn4*-Δ with the deletion of the *RPN4* gene, which encodes a key transcriptional regulator of proteasomal genes [45,46,47], an increase in the level of the large catalytic subunit of ribonucleotide reductase Rnr1p was also observed under normal conditions (Figure 1A,B), while the level of *RNR1* mRNA did not change significantly (Figure 1C). In addition, we confirmed that treatment with the DNA-damaging compound 4-nitroquinoline-1-oxide (4-NQO) resulted in an increased content of the small regulatory subunit Rnr2p in *rpn4*-Δ and *pre1–8* strains (Figure 1D,E), while the level of *RNR2* mRNA upon 4-NQO induction was not significantly different in all strains (Figure 1F). We have previously shown that Rpn4p is involved in the direct regulation of the expression of some genes of DNA repair systems [21,22]. The results indicate that Rpn4p does not affect *RNR* genes expression. In addition, the results indicate that under conditions of DNA-damaging stress, the ubiquitin–proteasome system is involved in the control of Rnr2p levels.

### 2.2. RNR Activity Is Elevated When Proteasome Function Is Impaired

Since proteasome dysfunction in yeast results in increased levels of at least two RNR subunits, we hypothesized that this could lead to increased enzyme activity. However, the presence of multilevel molecular mechanisms, including post-translational mechanisms controlling RNR activity and the involvement of the ubiquitin–proteasome system in the regulation of various intracellular pathways, makes the relationship between the quantities of subunits of the enzyme and its activity unclear. To indirectly assess RNR activity under conditions of impaired proteasome function, we determined the level of the enzyme-catalyzed reaction products, i.e., dNTPs, using specific real-time PCR according to a modified protocol described in [48]. 

**Figure 2:**
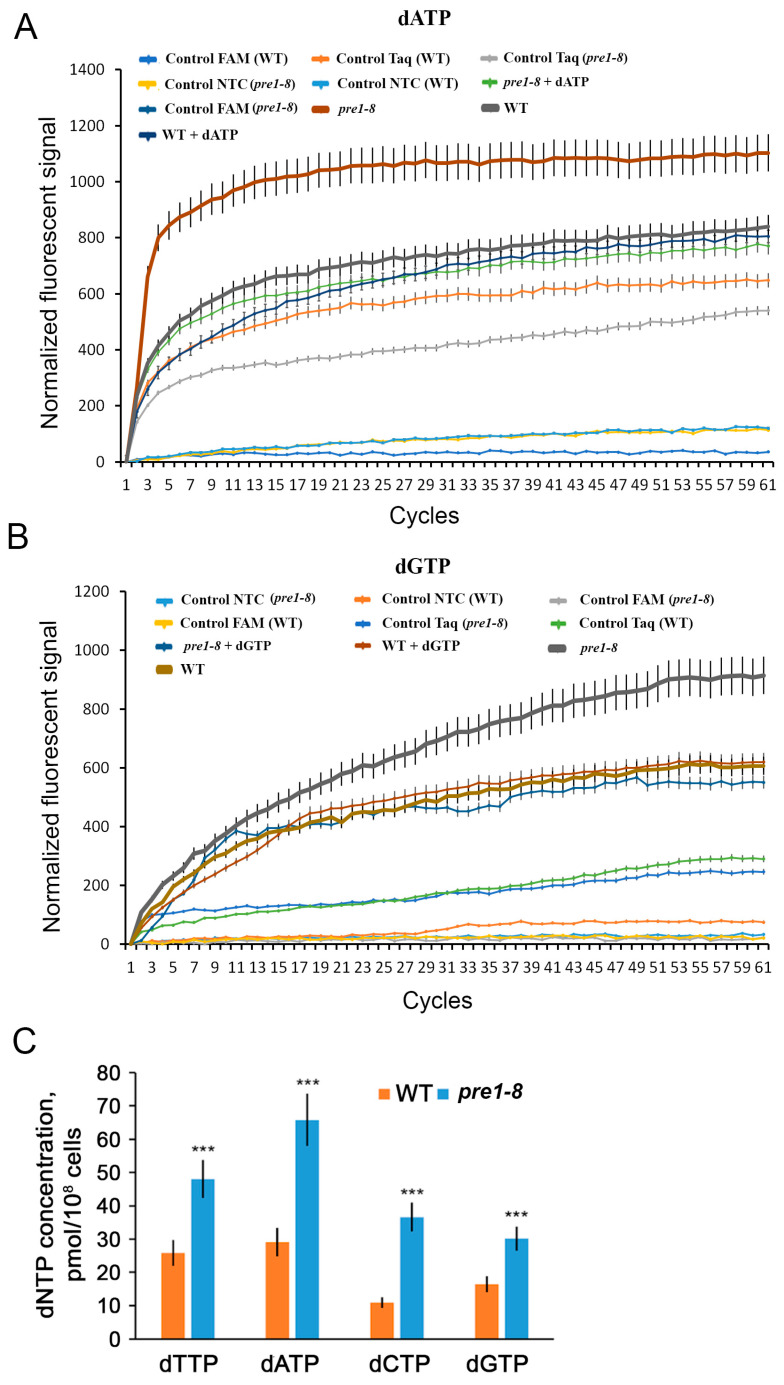
Disruption of proteasome function increases dNTP levels. The curves of accumulation of normalized fluorescent signal in the experiment, as well as in negative and positive controls for (**A**) dATP and (**B**) dGTP, are shown as examples. (**C**) Levels of dNTPs in WT and *pre1–8* yeast strains. Data represent mean ± SD (*n* = 9). Statistical significance: *** *p* < 0.001, according to Student’s *t*-test.

While using the original method for measuring dNTP concentration described in [48], we found a high variability of fluorescence signals between technical replicates of the same experiment. This variability results in negative values of normalized fluorescence units. We found that high data variability is associated with different initial fluorescence signal levels, which may be due to the low quality of the PCR reagents, consumables, or other technical reasons. To reduce the variability of the data, we propose to subtract the fluorescence signal value of the first cycle from the other cycles in the same reaction as the first step of data normalization. This operation allows us to obtain signal accumulation curves of the same shape as in [48]. We also found that a high total duration of the experiment leads to a decrease in the total fluorescence signal, which is apparently due to the photobleaching of the fluorescent dye FAM [49,50]. The reduction in the cycle time from 5 min to 10 s not only allowed us to reduce the total reaction time from 90 min to 12 min but also to reach the plateau of the fluorescence signal accumulation curve, i.e., to completely preserve the appearance of the experimentally obtained normalized curves as in the case of [48]. Moreover, we found that in the negative control without Taq polymerase, a significant signal is generated (Figure 2A,B), which contributes significantly to the total fluorescence signal. The presence of a signal in this control indicates the occurrence of non-enzymatic reactions that may lead to the hydrolysis of the probe. Given these data, in the second step of data normalization, the values of the controls without Taq are subtracted from the signals of the calibration curves as well as the curves for the quantification of dNTPs.

Using the optimized method, we determined the levels of dNTP content in the wild-type strain and in the *pre1–8* strain (Figure 2C). According to the data obtained, the levels of dNTPs differed in both strains, which may reflect the extent to which they are required for various cellular processes other than DNA replication and repair. The levels of all four dNTPs are higher in the mutant strain compared with the wild-type strain. Thus, decreased proteasome activity leads to increased levels of dNTPs, indirectly indicating increased RNR activity.

### 2.3. Inhibitors of Proteasome and RNR Exhibit Synergistic Effects in Yeast

Both proteasome and RNR inhibitors are used in tumor therapy [3,4,5,6,7,51]. In the course of therapy, tumor clones sensitive to these inhibitors are eliminated, and there are risks of the emergence of clones refractory to therapy [52]. One of the ways to fight refractory tumors may be combinatorial therapy using several therapeutic agents [15,16,17]. Given the similarity of molecular mechanisms of RNR regulation in yeast and mammals, and the fact that hydroxyurea inhibits RNR activity in both mammals [53] and yeast [54], it is important to answer the question whether yeast can serve as a simple model for screening combinations of proteasome and RNR inhibitor pairs.

**Figure 3:**
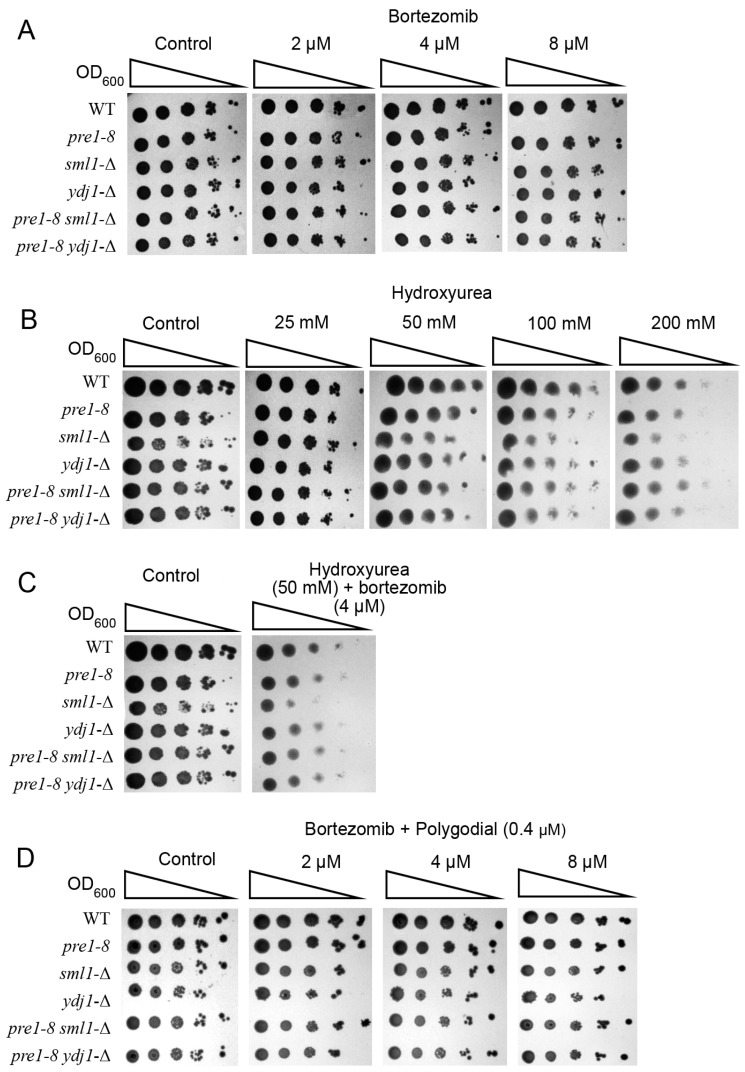
Evaluation of yeast strains’ resistance to the proteasome inhibitor bortezomib (**A**), the RNR inhibitor hydroxyurea (**B**), the combination of inhibitors (**C**) (the control images for panels B and C in Figure 3 are identical) (**D**) and the combination of bortezomib and polygodial, a cell permeabilizing agent.

**Figure 4:**
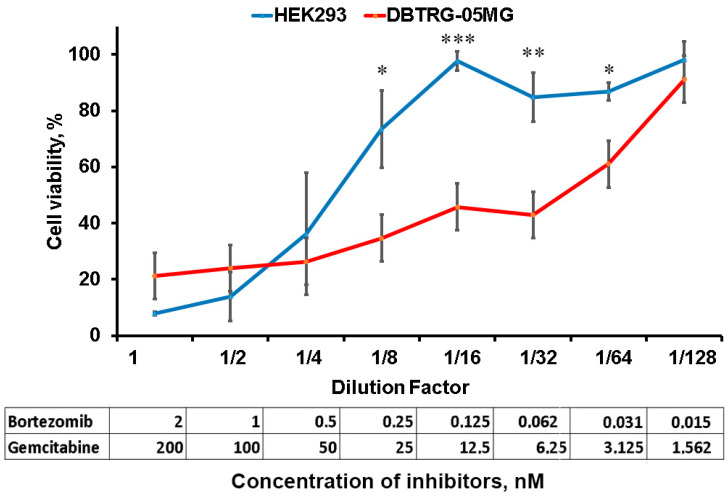
Survival of HEK 293 and DBTRG-05MG cell lines upon treatment with a combination of proteasome and RNR inhibitors. The data in the table represent the concentration of inhibitors in the corresponding dilution. Data represent mean ± SD (*n* = 3). Statistical significance: * 0.01 < *p* < 0.05, ** 0.001 < *p* < 0.01, *** *p* < 0.001, according to Student’s *t*-test.

Since the phenotypic inhibition of RNR [54] and proteasome [21] in yeast results in growth delay or death, the effectiveness of bortezomib and hydroxyurea was evaluated by the degree of growth suppression of yeast colonies (Figure 3). In the experiment we used not only wild-type strains and strains with impaired proteasome function but also strains carrying deletions of *SML1* (*sml1-*Δ) or *YDJ1* (*ydj1-*Δ) genes encoding regulators of RNR activity. *YDJ1* encodes the cytoplasmic co-chaperone Hsp70 involved in the stabilization of the RNR complex [55]. *SML1* encodes an allosteric inhibitor of RNR, which under the conditions of DNA damage is degraded by the ubiquitin-dependent pathway [56], which leads to RNR activation. At the first stage of the experiment, we determined the maximum concentrations of inhibitors that did not cause a significant negative effect on the colony formation of yeast (Figure 4A,B). In the case of bortezomib, no significant changes in the growth rate of yeast colonies were detected at all concentrations (Figure 3A). This phenotype is similar to that of tumor cells refractory to the action of proteasome inhibitors. In the case of hydroxyurea, the maximum concentration that did not cause colony growth inhibition was found to be 50 mM (Figure 3B). The use of a combination of inhibitors resulted in a significant inhibition of yeast colony growth and death, especially the mutant *sml1-*Δ (Figure 3C). 

However, proteasome inhibitors are known to penetrate poorly through the yeast cell envelope, and additional measures (the addition of 0.003% SDS to the nutrient medium or deletion of the *PDR5* transporter gene) are required to enhance their inhibitory effect. One could hypothesize that hydroxyurea may enhance the action of a proteasome inhibitor by increasing the permeability of the yeast cell membrane. To check this assumption, we performed a test for the resistance of yeast strains to bortezomib in the presence of the permeabilizing agent polygodial. Previously, we showed that polygodial enhances the inhibitory effect of proteasome inhibitors to a greater extent than the use of 0.003% SDS or a mutant strain with the deletion of *PDR5* transporter genes [21]. According to the results obtained (Figure 3D), no significant growth inhibition of all yeast strains was observed in the presence of polygodial and bortezomib at concentrations of 2, 4 or 8 µM. These data indicate that it is unlikely that hydroxyurea enhances the inhibitory effect of bortezomib by increasing cell permeability to bortezomib. Therefore, we can conclude that the addition of the RNR inhibitor allowed overcoming the phenotype of yeast resistance to bortezomib.

### 2.4. Inhibition of Proteasome and RNR Suppresses Glioblastoma Cell Growth

We next reproduced the inhibitory effect of the combination of proteasome and RNR inhibitors on the GBM cell line DBTRG-05MG. We first determined the IC50 separately for bortezomib and the RNR inhibitor gemcitabine in HEK293 (used as quasi-normal cells) and DBTRG-05MG cell lines (Table 1). Our results show that DBTRG-05MG is more resistant to each of the inhibitors used compared to HEK293.

We then used a mixture of inhibitors at concentrations that ensure at least 90% survival of the HEK293 cell line, and then prepared 2-fold dilutions. The initial concentration of bortezomib was 2 nM and gemcitabine was 200 nM. The survival of HEK293 and DBTRG-05MG when exposed to the initial mixture of inhibitors or their dilutions was assessed by resazurin staining (Figure 4). Our results suggest synergistic high toxicity of the original combination of inhibitors: HEK293 survival rate is 13.9% and DBTRG-05MG survival rate is 24%. As the inhibitor mixture is diluted, the survival of both lines increases, and at dilutions of 1/8 to 1/64, the survival of HEK293 is higher than that of DBTRG-05MG. Notably, at dilution 1/16, the maximum survival of HEK293 close to 100% is observed, whereas the survival of DBTRG-05MG is 45.8%. Thus, the combination of proteasome and RNR inhibitors is capable of being much more toxic to GBM cells than to quasi-normal HEK293 cells.

## 3. Discussion

We found that increased levels of RNR subunits due to impaired proteasome function leads to an increase in dNTP levels, which indirectly indicates an increase in enzyme activity. Moreover, it is shown that yeast can be used as a simple model for evaluating the synergistic antitumor effect of proteasome and RNR inhibitors.

Our data provide new evidence of the similarity between the mechanisms of RNR activity regulation in yeast and mammals. So, we have previously shown that the yeast subunit Rnr1 [23], as well as its orthologue in mammalian cells RRM1 [35], serves as a substrate of the proteasome. Since RRM2, the ortholog of the yeast Rnr2 subunit, is controlled in a cell cycle phase-dependent manner by the ubiquitin–proteasome system [34,57], we can assume that the yeast Rnr2 subunit also serves as a proteasome substrate under DNA-damaging stress. The overexpression of RNR subunits in mice causes an increase in the activity of the whole enzyme [58]. In our case, impaired proteasome function also leads to an increase in RNR activity. The decrease in the level of Rnr2p in the wild-type strain under the action of 4-NQO (Figure 1D,E), taking into account the fact that the enzyme functions as a tetramer consisting of two homodimers Rnr1/RRM1 and Rnr2/RRM2 [24], indicates a decrease in the level of the active enzyme content. This is in good agreement with the data on the severe delay in colony formation and reduced survival of wild-type yeast under the conditions of 4-NQO action [21,22]. Similarly, in mammals, various modes of transcriptional repression or targeting RRM2 result in the suppression of tumor growth, including GBM [44,59,60]. RNR catalyzes the synthesis of dNTP by a radical mechanism [24], which can be counteracted by the formation of reactive oxygen species. Since 4-NQO also induces oxidative stress [61], it is possible that the reduction in Rnr2p is a compensatory mechanism to reduce oxidative stress. Yeast strains with impaired proteasome function are apparently incapable of the negative regulation of Rnr2p, while they, like other organisms, are super-resistant to oxidative stress, which is associated with the overexpression of components of antioxidant systems [62,63,64,65].

One of the mechanisms of GBM resistance to chemotherapy may be the activation of membrane transporters belonging to the group of proteins responsible for multidrug resistance (MDR) [66]. The yeast genome encodes several transporters that provide MDR [67]. The inactivation of one of the most active transporters, Pdr5, is known to increase yeast sensitivity to various inhibitors, including proteasome inhibitors [68]. Thus, yeast with normally functioning cellular transporters can serve as a simple biochemical model of GBM refractory to therapy.

In combinatorial schemes of tumor therapy, proteasome inhibitors can be used together with therapeutic agents that damage DNA [69,70]. In mammalian cells, RNR serves as a substrate of the proteasome [34,35], so it can be assumed that the use of proteasome inhibitors results in the accumulation of the enzyme. Thus, the data obtained in the yeast model suggest that the use of proteasome inhibitors alone or their combinations with DNA-damaging compounds may be ineffective due to RNR hyperactivation. On the contrary, RNR inhibition enhances the efficacy of anticancer therapy [71,72]. Indeed, the combination of an RRM2 inhibitor and bortezomib has recently been shown to effectively suppress the growth of MM cells refractory to the action of bortezomib [41]. These data, as well as our findings, indicate the potential efficacy of the combined use of proteasome and RNR inhibitors against GBM.

Our data also indicate that the disruption of the function of RNR protein regulators may enhance the effect of inhibitors. Despite the fact that Sml1p is described as an RNR inhibitor, its deletion in the wild-type strain increases the sensitivity of the mutant strain to the synergistic action of proteasome and RNR inhibitors (Figure 3C). It should be noted that in the background of the mutation that reduces proteasome activity, the deletion of *SML1* does not result in increased sensitivity to the action of the combination of inhibitors (Figure 3C). Considering that the proteasome is involved in the control of activity of various regulatory proteins and signaling pathways, it is possible that in the case of *pre1–8,* there may be an indirect compensation of Sml1 dysfunction. No Sml1p orthologs have been found in mammals [73], but there is DNAJA1 ortholog Ydj1p, which is involved in positive regulation of yeast RNR activity. 

Another limitation of the study is that gemcitabine requires activation by cytidine kinase (dCK), which is expressed in mammalian but not yeast cells. Recently, a humanized yeast strain expressing dCK was obtained [74], allowing experiments with RNR inhibitors that require dCK-mediated activation. On the other hand, hydroxyurea is a drug that has long been used for cancer treatment [75], so yeast can still be used as a model to evaluate the molecular mechanisms of action of hydroxyurea and its combinations with other inhibitors.

Thus, our data indicate that, with certain limitations, yeast can serve as a simple biochemical model for the preliminary testing of combinations of existing and search for new proteasome and RNR inhibitors for GBM therapy. Further validation of the discovered combinations of inhibitors and more precise selection of their active doses on higher-level models of GBM, such as primary cells obtained from patients with tumors refractory to therapy, tumor organoids or mice with tumor xenografts, are certainly needed.

## 4. Materials and Methods

### 4.1. Strains and Cell Lines

Wild-type yeast strain BY4742 (MAT α; *his3*∆1; *leu2*∆0; *lys2*∆0; *ura3*∆0, Euroscarf, Oberursel, Germany), its derivative strain with reduced proteasome activity *pre1–8* (BY4742 MAT α; *his3*∆1; *leu2*∆0; *lys2*∆0; *ura3*∆0; *pre1–8*) [21], as well as derivatives of strain BY4741 (MAT a; *his3*∆1; *leu2*∆0; *lys2*∆0; *ura3*∆0, Euroscarf, Oberursel, Germany) and *pre1–8* with deletions of genes encoding RNR regulators, *sml1*-Δ (BY4741 *YML058W*::*LEU2*) [23], *ydj1-*∆ (BY4741 *YNL064C::LEU2*) [23], *pre1–8 sml1*-Δ (*pre1–8 YML058W*::*LEU2*) [23] and *pre1–8 ydj1-*∆ (*pre1–8 YNL064C::LEU2*) [23], were used in the experiments. 

HEK293 and DBTRG-05MG (ATCC no. CRL-2020) cell lines were cultured in Dulbecco’s modified Eagle’s medium (DMEM; PanEco, Moscow, Russia) with high levels of glucose containing 10% fetal bovine serum (Thermo Fisher Scientific, Waltham, MA, USA) and a mixture of penicillin and streptomycin (PanEco, Moscow, Russia).

### 4.2. Real Time PCR

Yeast cell culture grown for 16–18 h was diluted to an optical density of OD600 = 0.25 and grown for 4 h at 30 °C with constant shaking at 200 rpm. Cells were precipitated by centrifugation at 3000× *g* for 5 min. Cell pellet was disrupted with glass beads in a Precellys 24 homogenizer (Bertin Technologies, Montigny-le-bretonneux, France) at 6800 rpm, 3 cycles of 20 s in lysis buffer A (Evrogen, Moscow, Russia), and total RNA was isolated using an RNA Solo kit (Evrogen, Moscow, Russia). cDNA synthesis was performed using RevertAid H Minus reverse transcriptase (Thermo Fisher Scientific, Waltham, MA, USA) and oligo(dT) primer according to the manufacturer’s protocol. Relative mRNA level was estimated by real-time qPCR with SYBR Green I dye (Syntol, Moscow, Russia) on a CFX96 Touch™ real-time PCR detection system (Bio-Rad Laboratories, Hercules, CA, USA). The *ACT1* gene was used as a reference. Primary data were processed using CFX96 Maestro v2.3 Software supplied with the instrument and further analyzed in Microsoft Excel 2019 MSO (16.0.13328.20262) (Redmond, WA, USA). Oligonucleotides are summarized in Table 2.

### 4.3. Determination of dNTP Levels

Pools of dNTPs in yeast lysates were measured as previously described in [48] with modifications. Yeast cultures were grown to early log phase OD600 = 0.4–0.5. Cells were centrifuged and washed sequentially with nuclease-free water and 60% methanol. After the addition of 100% methanol, cells were lysed using acid-washed glass beads (0.5 mm, Sigma) in a Precellys 24 homogenizer (Bertin Technologies, Montigny-le-Bretonneux, France) at 6800 rpm, 6 cycles, 10 s for each cycle with pauses of 2 min on ice. Cell lysates were incubated for 30 min in a bath filled with copper-coated metal beads (4.5 mm diameter) pre-cooled to −80 °C. This bath is similar to the metal bath used in [76]. Cell extracts were then filtered using Amicon ultra 0.5 mL 3 K filters at 14,000 rpm for 20 min. The filtrates were heated at 98 °C for 3 min to denature the proteins. The denatured proteins were precipitated by centrifugation at maximum speed for 5 min. The supernatants were transferred to new tubes and evaporated at 72 °C for 3 h. The precipitates were resuspended in nuclease-free water and stored at −20 °C until use. The amount of dNTPs was measured by qPCR using an oligonucleotide matrix system and fluorescently labeled oligonucleotides by calibration curves. The control system for each dNTP included a control without dNTP (NDC), a control without matrix (NTC), a control without DNA polymerase (Taq) and a control without a fluorescence-activated probe (FAM). Fluorescence was detected in a CFX96 Touch™ real-time PCR instrument. Primary data were analyzed using Bio-Rad CFX Maestro v2.3 software. The analysis was then continued in EXCEL software 2019 MSO (16.0.13328.20262).

### 4.4. Determination of Yeast Cultures Resistance to Proteasome and Ribonucleotide Reductase Inhibitors

Yeast cell cultures were grown in complete YPD medium under constant shaking at 200 rpm for 16–18 h at 30 °C. The cultures were then diluted to an optical density of OD600 = 1. Then, a series of sequential 5-fold dilutions of yeast cell suspension was prepared and seeded on agarized nutrient medium with a proteasome inhibitor and/or hydroxyurea. In the control, strains were grown on medium without the addition of stress agents. The resistance of yeast strains to inhibitors was determined qualitatively by the rate of colony formation.

### 4.5. Western Blot Analysis

Yeast cell cultures transformed with plasmids encoding RNR genes were grown for 16–18 h on selective medium without uracil. The obtained cultures were diluted to OD600 = 0.25 and grown for 2 h at 30 °C. The cultures were divided into 2 parts, and one part was grown under normal conditions, and 4-NQO was added to the second part to the final concentration (1 мg/mL) and cultured for 4 h at 30 °C. The cells were precipitated by centrifugation at maximal speed at room temperature. Cell lysates were prepared by alkaline treatment as described in [77]. Lysates were separated by electrophoresis in SDS-PAGE polyacrylamide gel (7% in the case of Rnr1 or 10% in the case of Rnr2) and transferred to nitrocellulose membrane. The membranes were blocked with 5% skimmed milk and incubated first with primary mouse anti-HA antibodies (1:2000, Merck, KGaA, Darmstadt, Germany) and then with horseradish peroxidase-conjugated secondary anti-mouse antibodies (1:100,000, Jackson Immunoresearch Laboratories, West Grove, PA, USA). A signal was generated by incubating the membrane with ECL reagents (GE Healthcare Life Sciences, Piscataway, NJ, USA) and detected by exposure to photosensitive paper. Tubulin was used as a loading control. Tubulin was detected using primary rat monoclonal antibodies (1:2000, Abcam, Cambridge, UK) and horseradish peroxidase-conjugated secondary anti-rat antibodies (1:100,000, Abcam, UK) (1:100,000, Jackson ImmunoResearch Laboratories Inc., West Grove, PA, USA). The obtained images were analyzed using the ImageJ 1.52a program [78]. The signal intensities of the target proteins were normalized to the signal intensity of tubulin.

### 4.6. Cell Viability Assay

Resazurin staining was used to assess cell viability. For this purpose, 25,000 cells in a volume of 200 mL were seeded in a 96-well plate. The next day, inhibitors or equivalent volumes of DMSO were added to the medium and cells were incubated for 3 days. At the end of incubation, the cell culture medium was replaced with fresh medium containing 0.2 mM resazurin (100 μL/well), and cells were incubated for 4 hours. Absorbance was measured at 610 and 575 nm using signals read on a TECAN Spark multimode microplate reader (Tecan Trading AG, Männedorf, Switzerland). Background absorbance at 610 nm was subtracted from absorbance at 575 nm. Absorbance in wells without cells was used as a control. Cell viability was calculated relative to the DMSO-treated samples (taken as 100%) and presented as the mean value with standard deviation. Three biological replicates were used in each experiment. Each experiment was repeated twice.

## Figures and Tables

**Table 1:** Experimentally determined IC50 of proteasome and RNR inhibitors.

Cell Line	IC50 Bortezomib	IC50 Gemcitabine
HEK293	10.3 nM	1 µM
DBTRG-05MG	15.2 nM	24.4 µM

**Table 2:** Oligonucleotides used in the work.

Name	Nucleotide Sequence 5′ → 3′	Purpose	Reference
Primer- NDP1	CCGCCTCCACCGCC	Determination of dNTP levels	[48]
Probe- FAM-dTTP	6FAM/AGGACCGAG/ZEN/GCAAGAGCGAGCGA/BHQ		
Template- DT1-dTTP	TCGCTCGCTCTTGCCTCGGTCCTTTATTTGGCGGTGGAGGCGG		
Template- DT2-dTTP	TCGCTCGCTCTTGCCTCGGTCCTTTATTTATTTGGCGGTGGAGGCGG		
Probe- FAM-dATP	6FAM/TGGTCCGTG/ZEN/GCTTGTGCGTGCGT/BHQ		
Template- DT1-dATP	ACGCACGCACAAGCCACGGACCAAATAAAGGCGGTGGAGGCGG		
Template- DT2-dATP	ACGCACGCACAAGCCACGGACCAAATAAATAAAGGCGGTGGAGGCGG		
Probe- FAM-dGTP	6FAM/ACCATTCAC/ZEN/CTCACACTCACTCC/BHQ		
Template- DT1-dGTP	GGAGTGAGTGTGAGGTGAATGGTTTCTTTGGCGGTGGAGGCGG		
Template- DT2-dGTP	GGAGTGAGTGTGAGGTGAATGGTTTCTTTCTTTGGCGGTGGAGGCGG		
Probe- FAM-dCTP	6FAM/AGGATTGAG/ZEN/GTAAGAGTGAGTGG/BHQ		
Template- DT1-dCTP	CCACTCACTCTTACCTCAATCCTTTGTTTGGCGGTGGAGGCGG		
Template- DT2-dCTP	CCACTCACTCTTACCTCAATCCTTTGTTTGTTTGGCGGTGGAGGCGG		
RNR1-ex-top	GTTGCTGATATTTCCAACTTG	Real-time PCR	[23]
RNR1-ex-bot	CTATCTAGAGATGGAATAGTTG		
RNR2-ex-top	TGAAAAAGAGAGGTATGATG		
RNR2-ex-bot	GTCTGGTTTGTTCTTCAAATG		
ACT1-ex-top	CCTTCTGTTTTGGGTTTGGAATC		
ACT1-ex-bot	TGGAGCCAAAGCGGTGATTTCCT		

## Data Availability

All data supporting the findings of this study are available within the paper.
